# Endothelial nitric oxide signaling in cellular and systemic metabolism

**DOI:** 10.1016/j.redox.2026.104443

**Published:** 2026-10-18

**Authors:** Mauro Siragusa, Luke S. Dunaway, Eckhard Lammert, Ingrid Fleming, Brant E. Isakson, Miriam M. Cortese-Krott

**Affiliations:** a Institute for Vascular Signalling, Centre for Molecular Medicine, Goethe University, Frankfurt am Main, Germany; b CardioPulmonary Institute, Frankfurt am Main, Germany; c Department of Physiology, Tulane University School of Medicine, New Orleans, USA; d Heinrich Heine University, Faculty of Mathematics and Natural Sciences, Institute of Metabolic Physiology, Düsseldorf, Germany; e German Diabetes Center, Leibniz Center for Diabetes Research at Heinrich Heine University, Düsseldorf, Germany; f German Center for Diabetes Research (DZD e.V.), Neuherberg, Germany; g German Center for Cardiovascular Research (DZHK), Partner Site RheinMain, Frankfurt am Main, Germany; h Robert M. Berne Cardiovascular Research Center, University of Virginia School of Medicine, Charlottesville, USA; i Department of Molecular Physiology and Biophysics, University of Virginia School of Medicine, Charlottesville, USA; j Department of Pharmacology, Pharmacotherapy and Clinical Pharmacy, Institute of Pharmacy, Life Science Faculty, Eberhard Karls University of Tübingen, Tübingen, Germany

**Keywords:** *S*-nitrosation, Iron–sulfur clusters, Nitro-fatty acids, Angiocrine signaling, Lipid trafficking, Metabolic coordination

## Abstract

Endothelial cell-derived nitric oxide (NO) is best known for its role in the regulation of vascular tone. However, growing evidence shows that endothelial NO acts as a flux- and compartment-dependent redox regulator of cellular and systemic metabolism. NO directly modulates metabolic networks through reversible or irreversible chemical modification of iron centers, reactive cysteine and tyrosine residues as well as fatty acids. Mechanisms of NO-mediated regulation of cellular metabolism include the *S*-nitrosation of enzymes involved in glycolysis, oxidative phosphorylation and nucleotide biosynthesis as well as the control of iron and heme availability. NO-derived nitrating species additionally generate electrophilic nitro-fatty acids, which act on metabolic and redox transcriptional programs through reversible cysteine adduction. Moreover, specialized endothelial cells contribute to metabolic regulation across organs, for example lipid trafficking in adipose tissue, iron homeostasis in the liver and erythropoiesis in the bone marrow. Finally, tissue/organ-specific endothelial dysfunction may disrupt organ and systemic metabolic control through multiple mechanisms, raising new questions and therapeutic opportunities. Understanding how cell- and enzymatic source-specific NO signaling defines endothelial heterogeneity and function may provide a mechanistic framework to address organ-specific endothelial dysfunction in cardiometabolic disease.

## 1 Introduction

Nitric oxide (NO) plays a fundamental role in the regulation of both cellular and systemic metabolism by modulating metabolic enzyme activity, the subcellular trafficking of fatty acids, systemic lipid metabolism, inter-organ crosstalk and iron homeostasis [1], [2].

NO is enzymatically produced by the nitric oxide synthase (NOS) family of enzymes, which comprises two constitutively expressed enzymes, NOS1 (neuronal NOS; nNOS) [3] and NOS3 (endothelial NOS; eNOS) [4], and a third inducible isoform, NOS2 (inducible NOS; iNOS) [5]. Functional NOS enzymes function as homodimers, consisting of identical monomers that contain an N-terminal oxygenase domain binding heme, tetrahydrobiopterin and l-arginine, as well as a C-terminal reductase domain that binds flavin mononucleotide, flavinadenine dinucleotide and nicotinamide-adenine-dinucleotide phosphate (NADPH). These two domains are connected by a calmodulin-binding linker [6], [7]. In the isoforms with constitutive activity (eNOS and nNOS) calmodulin binding is Ca^2+−^dependent and reversible, and NO output is low and variable. iNOS, by contrast, binds calmodulin tightly even at basal Ca^2+^ concentrations, making the enzyme essentially Ca^2+^-independent. This isoform produces high NO fluxes (i.e. amount of NO that diffuses per unit area per unit time) over a prolonged period, i.e., as long as the protein is expressed, substrate is available and inhibitory modifications are lacking.

eNOS-derived NO is a key regulator of vascular function and homeostasis, maintaining endothelial integrity, inhibiting platelet activation and preserving vascular smooth muscle quiescence. Impaired eNOS activity or reduced NO bioavailability are linked to endothelial dysfunction, adhesion molecule expression and inflammation, contributing to vascular disease [8]. Genetic polymorphisms of eNOS associate with hypertension, coronary artery disease and stroke [9], [10], [11], [12], [13]. Mutations impairing the NO and soluble guanylyl cyclase (sGC) signaling also increase the risk of myocardial infarction [14], while genetic variants that enhance NO-cGMP signaling are protective against coronary and peripheral artery disease as well as stroke [15].

Endothelial cells are functionally heterogeneous across vascular beds [16], [17], [18], [19]. This is a fact highlighted in the single cell atlases that were used to identify transcriptional signatures of arterial, venous, capillary and lymphatic endothelial cells as well as between endothelial cells of different organs [17], [18]. Specialized endothelial cells, e.g. liver sinusoidal endothelial cells (LSECs), bone marrow sinusoidal endothelial cells and adipose capillary endothelium, carry out organ-specific metabolic functions that depend on local NO biology. This implies that eNOS-derived NO acts in a compartment- and tissue-specific manner and may be an important determinant of endothelial cell heterogeneity.

In this review we discuss how endothelial NO regulates metabolism through compartment-specific redox signaling shaped by local NO production, bioavailability and chemical reactivity. We first examine how endothelial NO regulates metabolism at the cellular level through direct regulation of mitochondrial respiration, *S*-nitrosation and nitration-dependent signaling, and control of iron and heme availability. We then describe how specialized endothelia coordinate metabolism across organs, focusing on iron homeostasis in the liver, lipid trafficking in adipose tissue and erythropoiesis in the bone marrow. Finally, we discuss how loss of endothelial NO bioavailability disrupts both cellular and systemic metabolic control and outline open questions and therapeutic perspectives.

## 2 Sources and targets of NO

### 2.1 Sources of NO

Steady-state NO concentrations generated by eNOS under physiological conditions are in the range of ∼100 pM to ∼5 nM [20], sufficient to activate sGC (EC50 ∼1–10 nM) and to drive selective *S*-nitrosation of reactive cysteines in specific target proteins. Under oxidative stress conditions, increased tetrahydrobiopterin oxidation or asymmetric dimethylarginine accumulation leads to reduced NO bioavailability and impaired metabolic signaling. Importantly, endothelial cells can also produce NO via alternative enzymatic and non-enzymatic pathways.

Under pro-inflammatory conditions, iNOS is transcriptionally upregulated via nuclear factor-κB (NF-κB) or other transcription factors in endothelial cells [21], [22], [23], [24], [25]. iNOS produces high amounts of NO (up to ∼1 μM) with output sustained over hours [26], [27], [28]. At these concentrations, NO engages in *S*-nitrosation and nitration reactions of a wider range of target proteins and fatty acids, including components of the insulin signaling cascade and mitochondrial respiratory chain [29], [30]. These redox-dependent modifications have been linked to vascular insulin resistance and impaired oxidative phosphorylation in experimental models of obesity and diabetes (Table 1).

**Table 1:** Genetic and pharmacologic evidence for iNOS-mediated metabolic effects Studies demonstrating that inducible nitric oxide synthase (iNOS, encoded by *Nos2*) contributes to skeletal muscle insulin resistance through S-nitrosation and tyrosine nitration of insulin signaling proteins.

#	Model/System	Manipulation	Tissue	Metabolic Phenotype	Mechanistic Insight	Refs.
1	High-fat diet (HFD) and ob/ob mice	Global Nos2^−/−^	Skeletal muscle	Protection from diet-induced insulin resistance	iNOS induction in obese muscle impairs PI3K–Akt signaling; prevented in Nos2^−/−^	(27)
2	Diet-induced obesity and ob/ob mice	iNOS inhibition; GSNO donor	Skeletal muscle	iNOS-linked insulin resistance; *S*-nitrosation of insulin-signaling proteins	*S*-nitrosation of IRβ, IRS-1 and Akt in obese muscle; reversed by iNOS inhibition	(29)
3	LPS-induced endotoxemia (rats and mice)	Global Nos2^−/−^; aminoguanidine	Skeletal muscle	Prevention of LPS-induced insulin resistance	iNOS-derived peroxynitrite drives tyrosine nitration of IRS-1	(31)
4	db/db diabetic mice; NO donors in vitro	Elevated iNOS-derived NO	Skeletal muscle; cultured cells	Akt inactivation in diabetic muscle	Direct *S*-nitrosation of Akt at Cys 224 inhibits kinase activity	(30)

Abbreviations: GSNO, S-nitrosoglutathione; HFD, high-fat diet; IRβ, insulin receptor beta subunit; IRS-1, insulin receptor substrate 1; LPS, lipopolysaccharide; NO, nitric oxide; PI3K, phosphatidylinositol 3-kinase.

Another endothelium-specific mechanism that shapes local NO bioavailability operates at the myoendothelial junction in resistance arteries. Endothelial cells in resistance arteries express α-globin, which forms a macromolecular complex with eNOS at myoendothelial junctions and scavenges locally produced NO, thereby restricting its diffusion to adjacent smooth muscle cells [32], [33]. Under hypoxia, however, endothelial α-globin functions as a nitrite reductase, whereby deoxygenated ferrous (Fe^2+^) heme reduces nitrite (NO_2_^−^) to NO while being oxidized to the ferric (Fe^3+^) state. This provides an oxygen-independent source of NO that supports hypoxia-induced vasodilation [34]. The heme-bound state of α-globin is required for NO scavenging, as iron deficiency shifts α-globin toward the apo form, reducing NO scavenging and increasing local NO bioavailability [32], [33], [35].

More recently, both α- and β-globin subunits have been shown to form a complex with eNOS in human resistance arteries, and genetic variants that alter globin expression or structure are associated with increased NOS-dependent vasodilation [36]. This mechanism represents a compartment-specific, iron-dependent regulator of endothelial NO bioavailability that is entirely distinct from classical NOS-driven synthesis and that connects endothelial iron status directly to NO signaling. In non-endothelial cell types, nitrite-derived NO formation transiently modulates mitochondrial respiration and improves perfusion in ischemic settings [8], [37], [38], [39], [40], [41].

There is also evidence to suggest that NO signals through NO-heme complexes rather than exclusively as dissolved gas [42], [43]. Together, these enzymatic and non-enzymatic systems generate NO fluxes that differ in magnitude, duration, subcellular localization and chemical reactivity, thereby producing distinct metabolic outcomes. Low-output eNOS-derived NO regulates homeostatic and selective metabolic signaling, whereas sustained iNOS-derived NO promotes nitrosative stress and metabolic dysfunction. Moreover, globin-mediated NO scavenging or nitrite reduction at the myoendothelial junction acts as a compartment-specific rheostat, tuning NO signaling according to vascular-bed identity, oxygen tension and iron availability.

### 2.2 Regulation of eNOS activity and NO output

The activity of eNOS is dynamically controlled by numerous protein-protein interactions, including heat shock protein 90, caveolin-1 as well as Ser, Thr or Tyr kinases such as Akt, AMP-activated protein kinase (AMPK), protein kinase A (PKA) and proline-rich tyrosine kinase 2 (Pyk2), which modulate enzyme conformation and phosphorylation state [44], [45], [46]. Phosphorylation on Thr495 prevents the binding of calmodulin [47], and phosphorylation on Tyr657 inhibits electron transfer, which prevents NO production [48], [49]. On the other hand, phosphorylation on Ser1177, Ser 615, Ser 633 and Tyr81 enhances activity under certain stimuli [50], [51], [52], [53]. The regulation and fine-tuning of these modifications determine cell- and tissue-specific eNOS activity and function under physiological and pathological conditions (for review see Ref. [54]).

Deficiency of tetrahydrobiopterin and post-translational modifications, such as acylation or *S*-glutathionylation, can *uncouple* eNOS, a process whereby the enzyme's electron transfer is diverted from l-arginine oxidation toward molecular oxygen, generating superoxide instead of NO, resulting in reduced NO bioavailability [55], [56], [57], [58]. This phenomenon has been linked to increased oxidative stress and endothelial dysfunction in early atherogenesis.

### 2.3 Targets of NO

Because of its chemical reactivity, NO exerts its biological effects by interacting with heme iron centers in target proteins leading to the formation of a *metal–nitrosyl complex* (defined as metal *nitrosylation*). The latter is the mechanism responsible for the activation of sGC, previously referred to as the “NO receptor” [59], [60], [61], [62]. NO can also react with cysteine residues in proteins, forming an *S*-nitroso group through *S-nitrosation*. Chemically, S-nitrosation proceeds via transnitrosation, N_2_O_3_-mediated mechanisms, or metal-catalyzed pathways [63], [64], [65], [66], [67]. This post-translational modification is regulated by enzymatic systems governing both addition and removal of NO groups; examples include GSNO reductase (GSNOR) and SNO-CoA Reductase 2 (SCoR2; also known as AKR1A1), which maintain redox and signaling homeostasis [68], [69], [70], [71], [72], [73]. Advances in redox proteomics and genetic approaches revealed that *S*-nitrosation is tightly regulated and controls protein function, subcellular localization and stability [74]. A wide range of cellular processes are modulated by *S*-nitrosation, including gene expression, cell survival, mitochondrial activity and cellular metabolism [75], [76], [77], [78].

In addition to metal nitrosylation and *S*-nitrosation, NO can contribute to cellular signaling through nitration reactions. Protein tyrosine nitration is generally not mediated by NO itself but by secondary reactive nitrogen species generated through NO-dependent oxidative chemistry, including peroxynitrite and nitrogen dioxide [79]. Nitration of tyrosine to 3-nitrotyrosine can alter protein activity, interfere with tyrosine phosphorylation and affect protein turnover, and is most commonly associated with conditions of increased NO and oxidant production rather than basal eNOS signaling. Several metabolically relevant proteins are susceptible to this modification.

NO-derived reactive species also modify unsaturated fatty acids to generate electrophilic nitro-fatty acids, providing a distinct link between NO, lipid and metabolic signaling. Fatty acid nitration occurs through non-enzymatic reactions involving nitrogen dioxide and is favored during oxidative inflammatory reactions, ischemia-reperfusion and gastric acidification. Conjugated linoleic acid is a preferential endogenous substrate for these reactions, and nitro-conjugated linoleic acid and its metabolites have been detected in human plasma and tissues [80]. Stable-isotope studies in healthy subjects further demonstrated that dietary nitrate or nitrite can give rise to circulating nitro-conjugated linoleic acid, establishing that nitro-fatty acids can be generated systemically *in vivo* [80]. The nitroalkene group renders nitro-fatty acids electrophilic, allowing reversible Michael addition to nucleophilic cysteine and histidine residues in proteins, a modification termed nitroalkylation [81]. This chemistry is distinct from *S*-nitrosation and expands the range of reversible cGMP-independent post-translational modifications derived from NO-dependent redox reactions. These nitroalkene–thiol adducts are reversible and are enzymatically removed by prostaglandin reductase-1, which acts as a nitroalkene reductase, and by β-oxidation, so that nitro-fatty acids behave as transient rather than terminal protein modifiers [82], [83]. Nitro-fatty acids regulate several pathways that link endothelial function to inflammation and metabolism [84].

Several of these effects are specific to the endothelium. In human endothelial cells, nitro-oleic acid suppresses inflammatory activation and the endothelial-to-mesenchymal transition [85] and activates Nrf2 through a Keap 1 cysteine-151-independent mechanism that distinguishes nitro-fatty acids from other electrophilic inducers [86]. Because nitroalkene adduction is reversible and is governed by membrane partitioning rather than aqueous diffusion, fatty acid nitration represents a second, lipophilic arm of reversible cysteine-directed NO signaling alongside S-nitrosation, with a distinct set of protein targets.

## 3 Regulation of endothelial cell metabolism by NO

Endothelial cells exhibit a distinctive metabolic phenotype characterized by predominant reliance on glycolysis for ATP production despite abundant oxygen availability [87], [88], [89], [90]. This glycolytic bias minimizes mitochondrial oxygen consumption and oxidative stress, while preserving oxygen for delivery to surrounding tissues. Mitochondrial respiration nonetheless remains essential for endothelial lipid transport, angiogenesis and the regulation of arterial tone [91], [92], [93], and the balance between glycolytic and oxidative metabolism is dynamically regulated in response to nutrient availability, redox state and signaling.

NO regulates endothelial metabolism through interconnected mechanisms that include nitration-dependent signaling (see above), direct reversible interactions with metal centers in metabolic enzymes, direct *S*-nitrosation of metabolic enzymes and control of iron metabolism and heme trafficking. The nature and magnitude of these effects likely depend on the source and flux of NO.

### 3.1 Regulation of mitochondrial respiration and oxygen utilization by NO

NO directly regulates mitochondrial oxygen utilization through reversible binding to cytochrome *c* oxidase (complex IV). NO interacts with the heme a_3_–Cu_B_ binuclear center and competes with O_2_, decreasing the apparent affinity of cytochrome *c* oxidase for oxygen; consequently, inhibition of respiration becomes progressively more pronounced as the local O_2_ concentration falls [94], [95]. This mechanism is particularly relevant in the vascular endothelium, where basal and agonist-stimulated eNOS-derived NO restrains mitochondrial O_2_ consumption at complex IV and thereby renders respiration sensitive to physiological changes in O_2_ availability [96], [97]. Reduced mitochondrial O_2_ consumption can in turn redistribute O_2_ toward non-respiratory oxygen-dependent processes and adjacent tissues, thereby modifying local oxygen gradients and oxygen-sensing pathways. Under hypoxic conditions, for example, NO-dependent inhibition of mitochondrial respiration increases the availability of O_2_ to prolyl hydroxylases and can alter the stability of hypoxia inducible factor-1α, while in endothelial cells endogenous NO also couples mitochondrial O_2_ handling to reactive oxygen species (ROS)- and AMPK-dependent signaling [89], [98]. The metabolic consequences depend strongly on NO flux and duration. Relatively low, transient eNOS-derived NO can reversibly adjust mitochondrial respiration to local O_2_ availability. In contrast, the higher and more sustained NO production associated with iNOS induction during inflammation can markedly suppress endothelial and vascular O_2_ consumption and, with prolonged exposure, cause more persistent inhibition of other respiratory components [96], [99]. Thus, the magnitude and duration of NO production, together with the local O_2_ concentration, determine the effects of NO on mitochondrial oxygen utilization and metabolic flux.

### 3.2 *S*-nitrosation as a direct metabolic switch

Endothelial cells retain considerable metabolic flexibility, despite their predominant reliance on glycolysis for ATP production, allowing them to adjust mitochondrial activity in response to changing physiological demands. Dynamic regulation of glycolytic flux allows endothelial cells to adapt to environmental changes, thereby linking metabolic state to vascular function. Although *S*-nitrosation has been reported for numerous glycolytic enzymes, only a few have been examined in detail (Table 2, Fig. 1).

**Table 2:** Metabolic enzymes undergoing *S*-nitrosation.

#	Metabolic enzyme	*S*-nitrosated site(s)	Refs
1	Glyceraldehyde-3-phosphate dehydrogenase (GAPDH)	Cys150	(100)
2	Phosphofructokinase, muscle isoform (PFKM)	Cys 351	(101)
3	Pyruvate kinase M2 (PKM2)	Cys 358	(102)
4	NADH dehydrogenase (Complex I), ND3 subunit	Cys 39	(103)
5	Cytochrome *c* oxidase (Complex IV), subunit II	Cys196; Cys200	(104)
6	Ribonucleotide reductase, large subunit (R1)	unknown	(105)

**Fig. 1:**
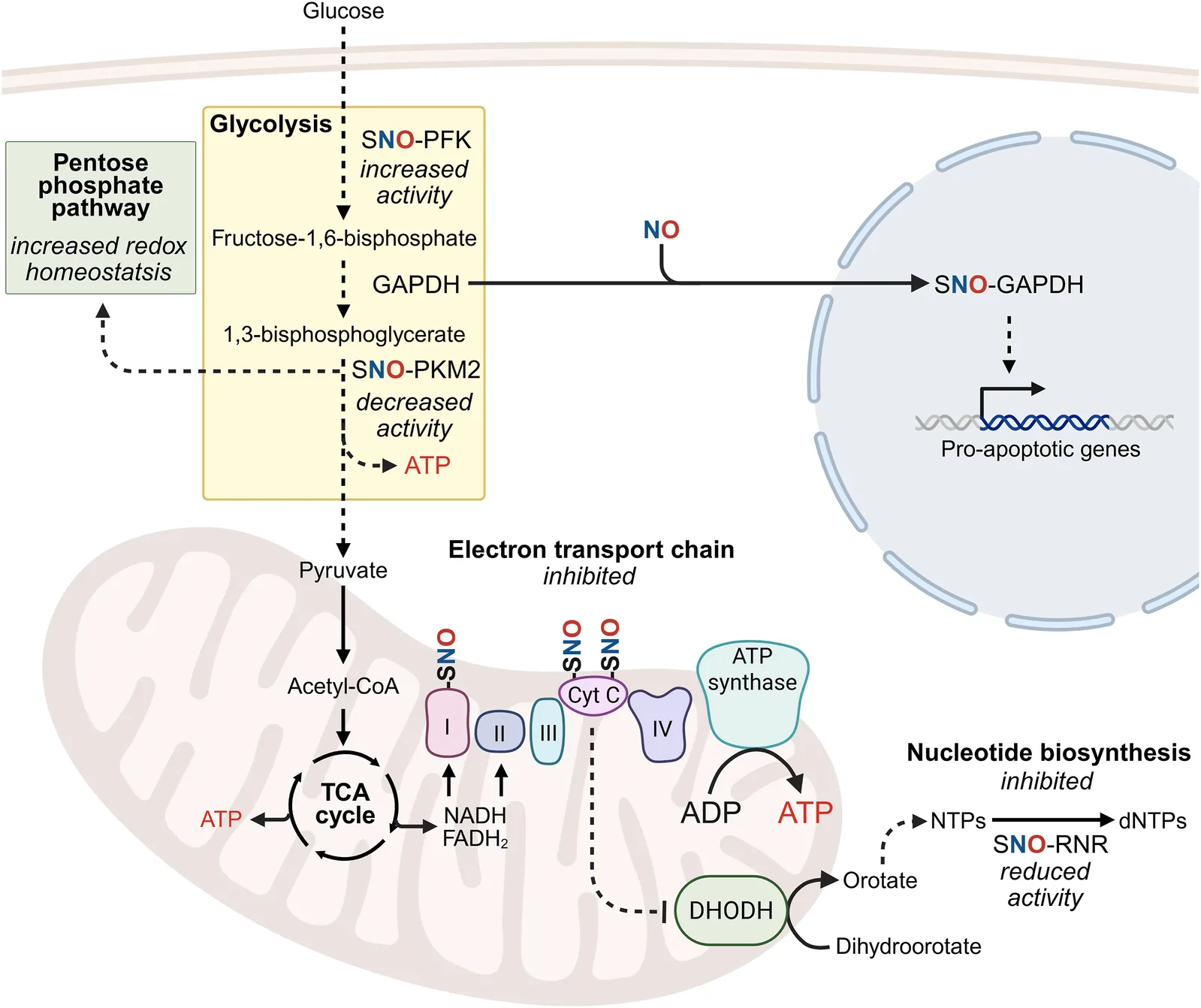
**Impact of *S*-nitrosation on key cellular metabolic pathways.***S*-nitrosation modulates enzymes across glycolysis, oxidative phosphorylation and nucleotide biosynthesis to dynamically control cellular metabolism and redox homeostasis. In glycolysis, modification of enzymes such as glyceraldehyde-3-phosphate dehydrogenase (GAPDH), phosphofructokinase (PFK), and pyruvate kinase M2 (PKM2) alters enzymatic activity, protein function and metabolic flux. Within mitochondria, *S*-nitrosation of respiratory chain components, including Complex I (NADH:ubiquinone oxidoreductase) and Complex IV (cytochrome *c* oxidase), regulates electron transport, adenosine triphosphate (ATP) production and reactive oxygen species generation, contributing to cytoprotection under stress. Additionally, nucleotide biosynthesis is influenced indirectly through respiratory inhibition and directly via *S*-nitrosation of ribonucleotide reductase (RNR), linking NO signaling to DNA and RNA precursor availability. Created with BioRender.com.

Early biochemical work established glyceraldehyde-3-phosphate dehydrogenase (GAPDH) as a prototypical *S*-nitrosation target. Exposure of cells to sodium nitroprusside leads to *S*-nitrosation, reversible inhibition of activity and increased endogenous ADP-ribosylation of GAPDH [106]. Mechanistically, *S*-nitrosated GAPDH acquires “moonlighting” functions, i.e. a signaling role distinct from its canonical metabolic activity in glycolysis. GAPDH *S*-nitrosated at the catalytic Cys150 (rat sequence, corresponding to human Cys 152) binds the E3 ligase Siah1, translocates to the nucleus and induces pro-apoptotic transcriptional programs. This is an elegant example of a metabolic enzyme co-opted by NO-dependent redox signaling [100].

Beyond GAPDH, further work has mapped specific glycolytic control points. In ovarian cancer cells, nNOS S-nitrosates the muscle/platelet isoform of phosphofructokinase at Cys 351, stabilizing the active tetramer, blunting feedback inhibition and sustaining glycolysis to support proliferation and metastasis in xenografts [101]. Pyruvate kinase M2 (PKM2), a rate-limiting glycolytic enzyme that contributes to the Warburg effect in cancer, is susceptible to *S*-nitrosation/oxidation [107], [108]. Detailed structural and enzymatic studies using cysteine point mutants demonstrated that *S*-nitrosation as well as oxidation regulate PKM2 oligomeric state and enzymatic activity. Oxidation and *S*-nitrosation of PKM2 at Cys358 inhibit enzyme activity, thereby shunting glucose away from glycolysis toward the pentose phosphate pathway, increasing NADPH production and supporting maintenance of the glutathione pool and endothelial redox homeostasis [102], [108]. By redirecting glycolytic flux toward the pentose phosphate pathway through PKM2 inhibition, NO also promotes the generation of ribose-5-phosphate, a critical substrate for de novo nucleotide and RNA synthesis. *S*-nitrosation of PKM2 has also been reported to be protective against acute kidney injury [72], [109].

In addition to glycolytic enzymes, *S*-nitrosation also regulates mitochondrial respiration at multiple levels of the electron transport chain and contributes to the acute control of mitochondrial respiration and cytoprotection (Table 2). Sustained exposure to NO at high concentrations inhibits cellular respiration, a phenomenon that is rapidly reversed by addition of the thiol-reducing dithiothreitol. These findings clearly implicate the *S*-nitrosation of critical respiratory chain thiols in the regulation of oxidative metabolism [96]. Complex IV itself has been reported to undergo *S*-nitrosation at the highly conserved Cys196 and Cys200 residues that can lock cytochrome *c* oxidase in low-activity states, resulting in reduced enzymatic activity [104]. Perhaps the clearest example of a defined *S*-nitrosation “switch”, however, occurs at complex I. During ischemia, Cys 39 in the ND3 subunit becomes exposed allowing *S*-nitrosation at the onset of reperfusion. This modification limits complex I activity, suppresses reactive oxygen species generation and oxidative damage, and confers cardioprotection *in vivo* [103].

*S*-nitrosation regulates nucleotide biosynthesis through both direct and indirect mechanisms. Nucleotide metabolism comprises the interconnected anabolic and catabolic pathways that govern the synthesis, interconversion and degradation of purine and pyrimidine nucleotides, thereby sustaining DNA and RNA biosynthesis, cellular energy transfer and nucleotide-dependent signaling processes. Direct, site-resolved *S*-nitrosation events on de novo nucleotide enzymes are less extensively characterized than those in glycolysis or oxidative phosphorylation, but two complementary mechanisms by which NO modulates nucleotide supply have been established (Table 2). First, by inhibiting cytochrome *c* oxidase, treatment with an NO donor acutely and reversibly suppresses dihydroorotate dehydrogenase, the inner-mitochondrial flavoprotein that couples pyrimidine synthesis to the ubiquinone pool [110]. Second, ribonucleotide reductase, the rate-limiting enzyme for deoxyribonucleotide formation, is directly susceptible to *S*-nitrosation. NO released from thionitrite inhibits ribonucleotide reductase through *S*-nitrosation of cysteines in the large R1 subunit along with effects on the radical-harboring small subunit [105].

### 3.3 Denitrosation

The reversibility of *S*-nitrosation is governed by enzymes catalyzing denitrosation reactions, which is equally important for metabolic regulation. Cellular denitrosation is mediated by enzymes that link the oxidation of NADH or NADPH to the reductive turnover of *S*-nitrosothiols. Among these, GSNO reductase (also known as alcohol dehydrogenase 5) is responsible for the majority of NADH-driven GSNO metabolism [111], [112], whereas SCoR2 functions predominantly as an NADPH-dependent reductase of both *S*-nitrosoglutathione and SNO-CoA in mammalian systems [113]. Beyond its enzymatic activity, SCoR2 has been shown to localize with components of the endoplasmic reticulum secretory pathway, where it modulates a transnitrosation signaling cascade. In this pathway, the cargo adaptor and small G protein secretion-associated and Ras-related protein 1 (SAR1) transfers an S-nitrosyl group to surfeit locus protein 4 (SURF4), which subsequently *S*-nitrosates proprotein convertase subtilisin/kexin type 9 (PCSK9), thereby suppressing its secretion. SCoR2 antagonizes this process by promoting denitrosation of PCSK9, effectively restoring its export [114]. Consistent with this regulatory role, genetic loss of SCoR2 in mice leads to diminished circulating cholesterol levels as a consequence of impaired PCSK9 secretion. Pharmacological inhibition of SCoR2 similarly reduces SNO-CoA metabolic flux across multiple tissues and confers protection from acute kidney injury, an effect attributed to enhanced *S*-nitrosation of PKM2 [71].

More recently, SCoR2 has emerged as a denitrosase that couples *S*-nitrosation dynamics to metabolic control in cardiovascular and metabolic disease [115], [116]. The depletion or pharmacologic inhibition of SCoR2 confers protection in experimental myocardial infarction by driving *S*-nitrosation-dependent remodeling of cardiac energy metabolism and redox balance. It also prevents diet-induced obesity and metabolic dysfunction-associated steatotic liver disease (MASLD) by suppressing lipogenesis and promoting oxidative pathways. Although the function of SCoR2 in endothelial cells remains largely uncharacterized, its broad regulatory effects suggest an important role in shaping endothelial *S*-nitrosation and NO-dependent regulation of metabolism.

### 3.4 Metabolic consequences of tyrosine nitration in endothelial cells

The peroxynitrite-mediated tyrosine nitration of insulin receptor substrate 1 impairs its interaction with phosphoinositide 3-kinase and reduces insulin-stimulated glucose uptake, while iNOS-dependent tyrosine nitration of the insulin receptor, insulin receptor substrate 1, insulin receptor substrate 2 and protein kinase B has been linked to hepatic insulin resistance during lipid overload [117], [118]. In endothelial cells, tyrosine nitration of mitochondrial manganese superoxide dismutase at Tyr34 inhibits its activity, promoting mitochondrial oxidative stress and impaired respiration [119]. Prostacyclin synthase represents another well-characterized endothelial target; nitration and inactivation of the enzyme under hyperglycemic conditions shifts prostanoid signaling toward vasoconstrictive and pro-inflammatory pathways [120], [121]. Thus, protein tyrosine nitration can couple altered NO/redox signaling to both metabolic signaling and endothelial dysfunction.

### 3.5 Signaling and metabolic effects of nitrofatty acids in endothelial cells

Nitroalkylation of the NF-κB p65 subunit suppresses NF-κB-dependent inflammatory gene expression and reduces endothelial adhesion molecule expression and leukocyte recruitment [122]. In human endothelial cells, nitro-oleic acid induces Nrf2-dependent cytoprotective genes and a broader heat-shock response [123], while also increasing eNOS expression and phosphorylation and inducing heme oxygenase-1 (HO-1) [124]. Nitro-fatty acids additionally regulate metabolic transcription through PPARγ; covalent modification of Cys 285 in its ligand-binding domain results in partial agonist activity, and administration of nitro-fatty acids to ob/ob mice lowers circulating glucose and insulin concentrations [125]. Nitroalkylation can also directly modify metabolic enzymes, as demonstrated by reversible modification and inhibition of GAPDH [81]. Nitro-conjugated linoleic acid was also shown to regulate transcriptional networks related to inflammation, oxidative stress, cell cycle control and hypoxic responses in primary human coronary artery endothelial cells [126]. Thus, nitrated lipids are cGMP-independent mediators that couple NO-dependent redox chemistry to local endothelial and systemic metabolic signaling. However, much of the mechanistic evidence derives from exogenous nitro-fatty acid treatment, and the concentrations, cellular sources and targets of endogenously generated nitro-fatty acids within individual vascular beds remain incompletely defined.

### 3.6 NO-mediated control of iron, iron-sulfur clusters and heme trafficking

Iron is essential for endothelial cell metabolism and function, serving as a cofactor in numerous proteins either as directly coordinated iron ions or as a constituent of iron-sulfur clusters and heme groups [127]. Ribonucleotide reductases utilize iron to synthesize deoxyribonucleotides [128]. Electron transport chain complexes I, II and III incorporate iron-sulfur clusters, while complexes II, III, IV and cytochrome *c* incorporate it into heme to mediate oxidative phosphorylation [129], [130]. Cytochrome P450 enzymes use heme to facilitate drug, steroid and eicosanoid metabolism [131]. When not chelated by iron-binding proteins, “*free”* iron catalyzes the formation of reactive oxygen species through the Fenton reaction and can trigger ferroptosis, a cell-specific iron-dependent form of cell death [132], [133]. Interestingly, lymphatic endothelial cell survival during development and after injury is regulated by ferroptosis, in contrast to blood endothelial cells, which are resistant to this form of cell death [134]. Iron transport and availability are therefore tightly regulated through a system of importers, exporters and storage proteins governed by iron regulatory proteins. The latter respond to intracellular iron status by post-transcriptionally controlling the expression of ferritin, ferroportin, transferrin receptor 1 and divalent metal transporter 1 to maintain iron homeostasis [135], [136].

Evidence for NO-mediated regulation of iron regulatory proteins in endothelial cells comes primarily from studies using exogenous NO donors, often at concentrations more representative of iNOS-driven nitrosative signaling than physiological eNOS-derived NO (Fig. 2). For example, in human umbilical vein endothelial cells, high concentrations of the *S*-nitrosothiol S-nitroso-N-acetylpenicillamine (SNAP) at (500 μM) prevent the upregulation of ferritin in response to iron loading through iron regulatory protein 1 activation [137]. Also, in bovine aortic endothelial cells exposed to combined hydrogen peroxide and the diazeniumdiolate NO donor (NONOate) treatment, TfR1 undergoes nitration and proteasomal degradation, reducing iron uptake [138]. In sheep pulmonary artery endothelial cells, high concentrations of SNAP (300 μM) induce HO-1, shifting iron distribution from heme to non-heme pools [139]. The iron-sulfur cluster disruption requires high NO concentrations consistent with iNOS rather than eNOS activity. Whether endogenous eNOS/iNOS-derived NO regulates these processes under physiological conditions *in vivo* remains unresolved. Studies in non-endothelial cells demonstrated that NO donors disrupt the 4Fe-4S cluster in iron regulatory protein 1, converting it to its mRNA-binding apo form, while stabilizing iron regulatory protein 2 against proteasomal degradation in fibroblast and cancer cell lines [140], [141], [142]. Together these mechanisms shift iron availability toward increased intracellular iron.

**Fig. 2:**
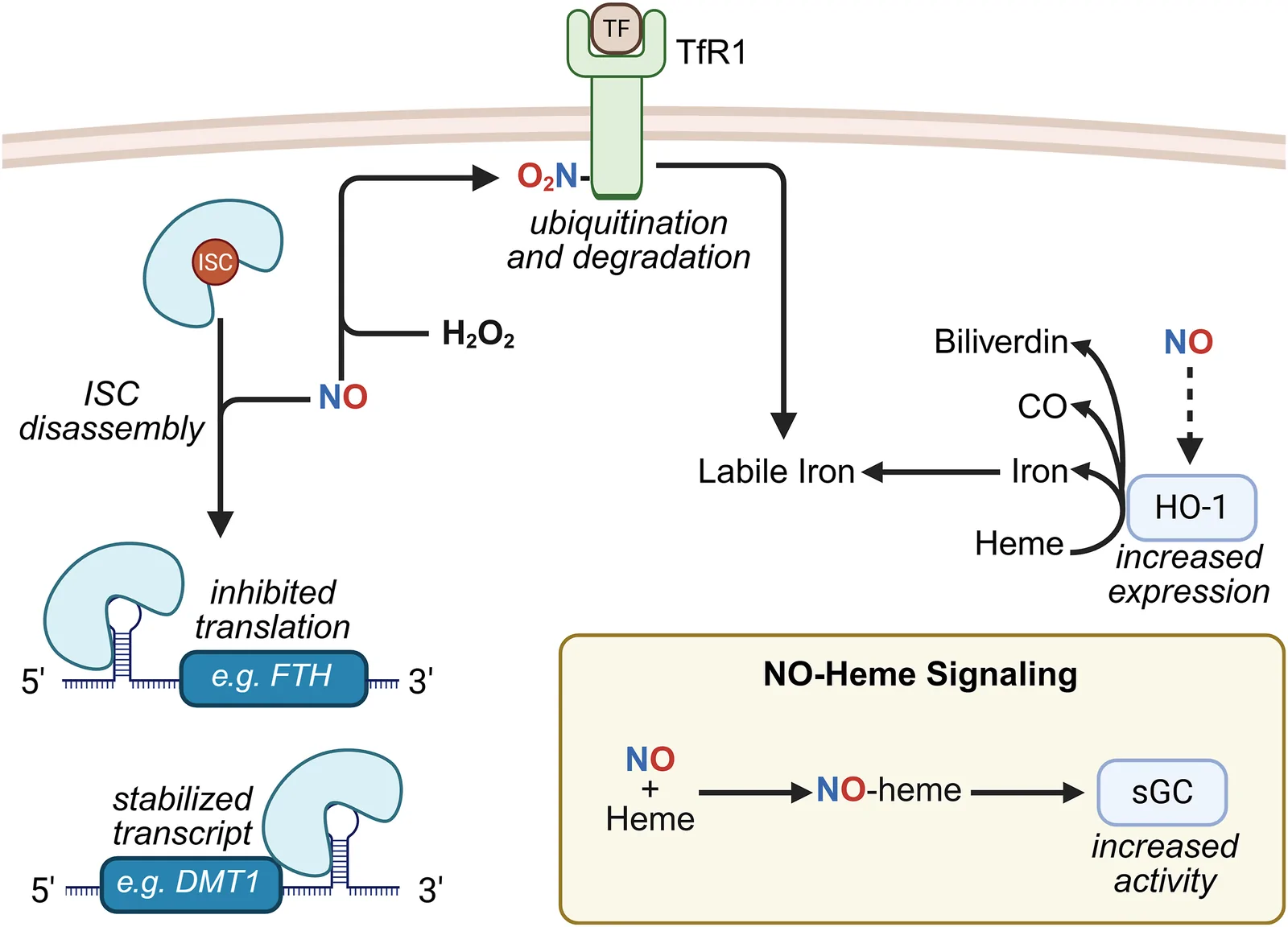
**Nitric oxide-mediated regulation of iron transport.** High concentrations of nitric oxide (NO) disrupt iron-sulfur clusters (ISC) in iron regulatory protein 1 (IRP1) and increase expression of heme oxygenase-1 (HO-1). Under oxidative conditions, the transferrin receptor (TfR1), which mediates iron import, is nitrated and subsequently ubiquitinated and degraded. Together, these processes facilitate the redistribution of iron between cellular compartments. NO also directly interacts with heme, promoting its insertion into hemoproteins such as soluble guanylyl cyclase (sGC). TF: Transferrin; FTH: Ferritin heavy chain; DMT1: Divalent metal transporter 1. Created with BioRender.com.

Interestingly, both stored and labile iron vary substantially among endothelial subtypes and influence endothelial identity [35]. Work in both mouse and human endothelial cells demonstrates that lymphatic endothelial cells have much higher levels of labile and stored iron than arterial endothelial cells in the microvasculature and iron chelation in vitro shifts lymphatic endothelial cell phenotype toward an arterial identity [35]. Since NO regulates multiple proteins involved in iron metabolism, differences in NO flux and compartmentalization across vascular beds may contribute to iron-dependent endothelial phenotypes. This possibility warrants further investigation as a potential determinant of endothelial heterogeneity.

Beyond its effects on iron pools, NO directly regulates heme trafficking and hemoprotein assembly. Approximately half of all iron-dependent enzymes are hemoproteins [127]. Early studies demonstrated that NO-heme complexes can be transferred between hemoproteins to activate sGC [143], and recent evidence shows that NO promotes heme insertion into apo-sGC, thereby facilitating assembly of its own receptor [144]. This reflects a broader mechanism by which NO increases labile heme and facilitates heme insertion into apo-hemoproteins [145]. The effects of NO reveal a biphasic concentration-dependent relationship [146], [147]. Thus, low NO concentrations facilitate heme insertion, whereas high concentrations decrease heme-bound protein [146], [147]. These findings support the hypothesis that NO signals partly through NO-heme complexes [42], [43], providing a mechanism for NO signaling in heme-rich environments where dissolved NO would otherwise be rapidly scavenged.

Taken together, NO-dependent regulation of iron homeostasis and heme trafficking has three important implications for endothelial function. First, by controlling iron regulatory protein activity and iron partitioning among the cellular pools, NO may also modulate iron availability for incorporation into metabolic enzymes, indirectly regulating their activity. Second, by promoting heme insertion into apo-sGC, NO signaling is self-reinforcing, as enhanced sGC assembly increases cellular responsiveness to subsequent NO signals. Third, endothelial iron status, which varies across vascular beds and is itself modulated by NO, may influence endothelial phenotype and metabolic function, linking iron biology to endothelial heterogeneity. Since NO flux determines both heme insertion into metabolic hemoproteins (low concentration/flux) and iron-sulfur cluster integrity in iron-dependent enzymes (at high concentration/flux), “free” iron and the presence or absence of heme in apo-hemeproteins become active determinants of how NO signals are propagated and “interpreted” within the endothelial cell.

### 3.7 How does NO flux and compartmentalization shape endothelial metabolism?

The examples discussed above indicate that the metabolic consequences of endothelial NO signaling depend on the source, bioavailability and the subcellular compartmentalization of NO rather than on NO concentration alone. Basal eNOS activity generates low levels of NO that can activate heme-containing targets and *S*-nitrosate specific cysteine residues in metabolic enzymes. Through these mechanisms, eNOS-derived NO may also modulate iron partitioning and heme insertion, thereby supporting adaptive metabolic responses, including redox homeostasis and mitochondrial regulation. The functional consequences of *S*-nitrosation of metabolic enzymes likely depend on the microenvironment of the modified cysteine as well as the prevailing cellular NO-thiol redox state. Denitrosation by GSNOR and SCoR2 maintains the specificity and reversibility of *S*-nitrosation, integrating NO signaling into the broader cellular redox landscape. In contrast, induction of endothelial iNOS under inflammatory conditions can lead to the sustained generation of high levels of NO that exceed the threshold for selective redox signaling and elicit broader nitrosative modifications of metabolic and signaling proteins. Such conditions may also favor protein nitration and the generation of electrophilic nitro-fatty acids, extending the metabolic consequences of NO beyond *S*-nitrosation.

Taken together, iron availability, heme status and the balance between *S*-nitrosation and denitrosation determine which metabolic enzymes are subject to NO-dependent regulation at any given time. Because endothelial cells across vascular beds differ substantially in iron content, eNOS activity/expression, and denitrosase expression, this flux- and compartment-dependent NO-mediated regulation may also help shape the metabolic identity of arterial, venous, capillary, and sinusoidal endothelial cells and thus determine intrinsic endothelial heterogeneity.

## 4 Systemic regulation of metabolism by endothelial cell-derived NO

Beyond its direct effects on endothelial cell metabolism, endothelial cell-derived NO coordinates systemic metabolic homeostasis through paracrine and angiocrine signaling to adjacent parenchymal cells. Endothelial cell-derived NO is known to regulate vasodilation and microvascular perfusion in all tissues. Accumulating evidence suggests that NO signaling regulates substrate delivery and angiocrine factor release from vascular and non-vascular endothelial cells, e.g. sinusoidal endothelial cells, endocardial endothelial cells, thereby shaping the metabolic activity of neighboring cells, tissues and potentially the organism as a whole. Yet an important question remains: to what extent do the source, magnitude, duration and subcellular localization of NO signals constitute defining features of endothelial functional heterogeneity and contribute to metabolic regulation?

### 4.1 Divergent roles of eNOS and iNOS in systemic metabolic homeostasis: evidence from murine models

Genetic models targeting eNOS and iNOS provide complementary insights into how NO and redox state influence metabolic outcomes *in vivo* (Table 1, Table 3). While eNOS deletion has helped to elucidate the metabolic consequences of low NO output, iNOS deletion has clarified the contribution of sustained, high-output NO production during inflammatory or metabolic stress.

**Table 3:** Genetic and pharmacologic murine models of eNOS regulation.

#	Model	Refs
1	Global Nos3^−/−^ hypertension	(148)
2	Nos3^−/−^ metabolic syndrome	(149)
3	eNOS and mitochondrial biogenesis	(150)
4	Endothelial IRS-2^−/−^ (insulin delivery)	(151)
5	Cav1^−/−^, loss of caveolae	(152)
6	Cav1^−/−^, eNOS up-regulation	(153)
7	eNOS S1176D/SA knock-in	(154)
8	eNOS Y656F knock-in	(102)
9	eNOS uncoupling, tetrahydrobiopterin	(155)
10	eNOS overexpression	(156)

Four independent global eNOS knockout strains have been generated [148], [157], [158], [159]. All of these eNOS^−/−^ mouse models develop hypertension but they differ in sex-dependent blood pressure regulation and protection from acute myocardial infarction (discussed elsewhere [160], [161], [162]). Studies using the Shesely eNOS^−/−^ mice have shown that eNOS deficiency results in insulin resistance, impaired glucose tolerance, fasting hyperinsulinemia and hyperlipidemia [149]. Hyperinsulinemic-euglycemic clamp studies comparing Huang eNOS^−/−^ and nNOS^−/−^ mice established that eNOS is the dominant isoform involved in metabolic regulation. Indeed, the lack of eNOS causes insulin resistance in both the liver and peripheral tissues, whereas nNOS deficiency produces only peripheral insulin resistance [163]. These effects are mainly related to changes in insulin signaling cascade in disease conditions [164].

The role of eNOS as downstream effector of insulin signaling is complex. In cell culture, insulin was initially described as activating eNOS and increasing NO production via Akt activation and phosphorylation of eNOS on Ser1177 [165]. However, while the acute stimulation of primary endothelial cells or isolated arterial vessels with physiological insulin concentrations does elicit a robust phosphorylation of eNOS on Ser1177, it does so without increasing cGMP levels or inducing rapid NO-dependent vasorelaxation. Insulin has also been shown to promote phosphorylation of eNOS on Tyr657 (human sequence; Tyr656 in mice) through the redox-sensitive kinase Pyk2, an event that inhibits eNOS enzymatic activity. Thus, when insulin elicits phosphorylation at both Ser1177 and Tyr657, Tyr657 phosphorylation appears to dominate functionally by impairing electron transfer through the flavin mononucleotide-binding domain, resulting in reduced eNOS activity [48], [166], [167]. Consistent with this model, endothelial cell-specific expression of a dominant-negative human insulin receptor in mice results in endothelial dysfunction, increased oxidative stress and reduced NO bioavailability [168]. Moreover, endothelial-specific deletion of Irs 2, which impairs insulin-induced eNOS phosphorylation, reduces capillary recruitment and attenuates skeletal muscle glucose uptake despite intact parenchymal insulin receptor function, demonstrating that endothelial NO production is required for normal insulin-dependent substrate delivery [151]. Notably, selective amplification of insulin signaling in the endothelium *in vivo* activates an Akt–Nox2–superoxide pathway that upregulates Pyk2, leading to increased phosphorylation of murine eNOS on Tyr656 and consequent reductions in eNOS activity and NO bioavailability [169]. Mice carrying a non-phosphorylatable Tyr656 F-eNOS mutant on an apolipoprotein E-knockout background demonstrate improved endothelial redox homeostasis and are protected against atherogenesis [75], [102], [170]. There also seems to be a clear explanation for the fact that numerous studies looking at insulin signaling in cultured cells were associated with increased NO production and eNOS activation. That is because the expression of Pyk2 is quickly downregulated and barely detectable in multi-passaged cells [48].

Very few metabolic studies were carried out on iNOS-deficient mice, but they are protected from diet-induced insulin resistance and preserve insulin signaling in skeletal muscle. These effects were attributed to an iNOS deficiency-dependent reduction in *S-*nitrosation and nitration of insulin signaling intermediates. This is consistent with the nitration-dependent mechanisms discussed earlier, and limits nitrosative inhibition of mitochondrial respiratory components [27], [28], [29].

One important limitation of these studies, however, is that they were carried out in mice with eNOS, nNOS or iNOS mutations in all tissues. These findings therefore require confirmation in cell type-specific models that can distinguish the effects of NO source, magnitude, and cellular context.

### 4.2 Liver sinusoidal endothelium: iron sensing, hepcidin regulation and MASLD

LSECs are highly specialized endothelial cells that line the hepatic sinusoids and constitute the primary interface between portal blood and hepatic parenchyma. Their distinctive morphology, which is characterized by transcellular fenestrae, the absence of a basement membrane and high endocytic capacity, facilitates bidirectional exchange of substrates, lipoproteins and signaling molecules between sinusoidal blood and hepatocytes [171]. A central role has been attributed to eNOS-derived NO in LSECs for maintaining sinusoidal vasodilation, preserving hepatic stellate cell quiescence and thereby preventing intrahepatic vasoconstriction and fibrosis [172], [173].

Beyond vascular regulation, LSEC-derived NO supports hepatocyte homeostasis through mechanically induced angiocrine signaling. For example, eNOS-dependent hepatic vasodilation drives the expression of interleukin-6, hepatocyte growth factor and likely Wnt2a and Wnt9b as well to enable hepatic regeneration [174], [175], [176]. Conversely, the loss of eNOS-sGC signaling in LSECs reduces fenestration, promotes stellate cell activation and impairs the angiocrine support of hepatocyte function [174], [177], [178], [179], [180], [181]. Under pro-inflammatory conditions, LSECs have also been shown to express iNOS [180], [181], which may contribute to LSEC capillarization and hepatic fibrosis [182], [183], [184].

Another important paracrine function of LSECs within the liver is iron sensing and regulation of iron storage in the hepatocytes. In response to intracellular iron accumulation, LSECs upregulate and secrete bone morphogenetic proteins 2 and 6 (BMP2/6), which in turn activate the expression of hepcidin in adjacent hepatocytes [185], [186], [187], [188], [189]. Hepcidin then binds to ferroportin, thus restricting iron release from liver stores [190], [191]. Although mitochondrial ROS has been identified as a key regulator of this pathway, the role of NO remains unclear [185]. While iNOS-derived NO does not appear to contribute to LSEC-mediated control of hepcidin expression, the importance of eNOS-derived NO remains to be investigated [192].

In MASLD, LSECs undergo profound phenotypic alterations that include the disruption of both vascular and metabolic homeostasis. Capillarization, defined by loss of fenestrations, development of a continuous basement membrane, increased oxidative stress and pro-inflammatory activation, is accompanied by reduced eNOS expression and activity [171], [193]. Similar changes occur during aging in a process termed pseudo-capillarization [194]. Capillarized LSECs lose their angiocrine support of hepatocyte function, fail to suppress hepatic stellate cell activation and thereby facilitate fibrosis progression and portal hypertension. In MASLD, saturated fatty acids increase the expression of Sema3A, an antiangiogenic factor and neuropilin-1 (vascular endothelial growth factor [VEGF] co-receptor) antagonist [195], [196], [197]. This reduces endothelial fenestration through a neuropilin-1 and cofilin-1-dependent mechanism and increases hepatic steatosis [197]. Interestingly, the Shesely eNOS^−/−^ mice show increased hepatic steatosis [149]. Whether these effects are partially mediated by decreased VEGF-induced eNOS signaling remains to be investigated.

LSEC dysfunction also impairs hepatic insulin signaling and promotes lipid accumulation, contributing to disease progression from simple steatosis to metabolic dysfunction-associated steatohepatitis [174], [178], [198]. Whether the disruption of LSEC iron sensing in MASLD contributes to the iron dysregulation frequently observed in this condition remains an important open question.

Endothelial dysfunction in MASLD extends beyond the liver. Patients with MASLD exhibit systemic endothelial dysfunction characterized by impaired flow-mediated vasodilation, increased arterial stiffness and elevated cardiovascular risk [199]. Loss of LSEC-derived NO and altered angiocrine factor profiles may link hepatic endothelial dysfunction to systemic vascular disease, though whether this reflects circulating factors, shared pathogenic mechanisms or direct inter-organ communication remains to be determined [200].

### 4.3 Endothelial regulation of lipid trafficking in adipose tissue

Microvascular endothelial cells in adipose tissue regulate fatty acid uptake and lipid trafficking into the tissue through expression of lipid transporters, including CD36, a non-esterified fatty acid transporter [201]. Recent work has demonstrated that CD36 in adipose tissue microvascular endothelial cells is directly regulated through *S*-nitrosation by eNOS-derived NO, providing a defined mechanism by which vascular bed-specific NO signaling controls tissue lipid uptake [202].

NO donors and nitrosothiols prevent lipid accumulation through *S*-nitrosation of CD36 at critical cysteine residues Cys 3 and Cys 466, two that are normally post-translationally modified by palmitoylation to promote trafficking to the plasma membrane. *S*-nitrosation of CD36 prevents palmitoylation thus prevents plasma membrane translocation, redirecting CD36 toward the endoplasmic reticulum and reducing lipid uptake capacity. This competitive post-translational regulation provides a mechanism by which eNOS-derived NO directly antagonizes lipid-dependent trafficking signals in adipose microvascular endothelial cells.

The relationship between NO and CD36 was validated *in vivo* using endothelial-specific caveolin-1 knockout (Cav1^−/−^) mice, which generate more NO than wild-type mice [153], [202]. Increased NO production resulting from the disinhibition of eNOS, usually exerted by Cav1, promoted CD36 retention in the endoplasmic reticulum and decreased lipid droplet accumulation. This phenotype was reversed by NOS inhibition, demonstrating that physiological levels of eNOS-derived NO limit endothelial lipid uptake through the post-translational modification of CD36. These mechanisms have important implication for endothelial cell metabolism, as endothelial dysfunction, i.e. the loss of eNOS-derived NO, would promote the plasma membrane targeting of CD36 to increase lipid uptake and fatty acid oxidation. This promotes endothelial mitochondrial dysfunction and aggravates the impaired acetylcholine-mediated vasodilation in small mesenteric arteries exposed to non-esterified fatty acids. Changes in endothelial CD36 activity also affect circulating plasma lipids and while endothelial cell Cav1^−/−^ mice have elevated plasma lipids, the opposite is the case in endothelial eNOS^−/−^ mice [202]. It is not clear whether this mechanism operates across multiple vascular beds and whether endothelial lipid uptake coordinates systemic lipid distribution. Reduced eNOS activity, as it occurs, for example, in capillarized LSECs in MASLD, could increase CD36-mediated hepatic lipid uptake and thereby contribute to steatosis.

Electrophilic nitro-fatty acids add a further, lipid-based layer of systemic metabolic regulation. They can be esterified into triacylglycerides and complex lipids, forming a storage and transport pool that is mobilized by lipolysis and distributed systemically [203], [204], and their formation is supported by dietary nitrite and nitrate [205]. *In vivo*, nitro-oleic acid reverses obesity-induced hepatic steatosis and protects against steatosis and fibrosis in steatotic liver disease [206], [207]. It accumulates in adipose tissue [208] and regulates intestinal lipid absorption [209], while also enhancing mitochondrial metabolism and ameliorating heart failure with preserved ejection fraction [210]. Additionally, nitro-oleic acid lowers blood pressure through inhibition of soluble epoxide hydrolase [211]. Notably, oral 10-nitro-oleic acid (CXA-10) is well tolerated and produces measurable pharmacodynamic responses in obese human subjects, although efficacy endpoints across trials have so far been mixed [212].

### 4.4 Role of bone marrow endothelium-derived NO in erythropoiesis

Erythropoiesis occurs in specialized microanatomical structures within the bone marrow called erythroblastic islands, which consist of a central macrophage surrounded by developing erythroblasts at different maturation stages. These islands are intimately associated with bone marrow sinusoidal endothelial cells, and the spatial relationship changes dynamically during erythroid maturation. Early erythroblast development occurs in regions distant from sinusoids, but as erythroid cells mature, the islands migrate toward the sinusoidal endothelium. Ultimately, mature erythrocytes cross the endothelial barrier and are released into the circulation through a process requiring coordinated endothelial-erythroid interactions.

Emerging evidence suggests that the NO-sGC-cGMP pathway regulates multiple steps of erythroid maturation. Of the two sGC isoforms, i.e. GC-1 (α1β1) is expressed in erythroid cells and red blood cells, while GC-2 (α2β1) expression is restricted to vascular and neuronal tissues [213], [214], [215]. Early work in human erythroid cell lines established that the sGC α-subunit is preferentially expressed in erythroid cells expressing γ-globin, and that sGC activators and cGMP analogs induce γ-globin expression in K562 erythroleukemic cells and in primary erythroblasts from normal subjects and β-thalassemia patients [213]. Later it was shown that hydroxyurea induces γ-globin in K562 cells and human erythroid progenitors through an NO-dependent mechanism sensitive to sGC inhibitors and reproduced by the NO donor S-nitrosocysteine [216]. Direct pharmacological stimulation of sGC with BAY 41-2272 similarly induces γ-globin and fetal hemoglobin production in erythroleukemic cells [217]. In vivo, sGC overexpression in erythroid progenitors promoted erythropoiesis, increasing red cell counts and hemoglobin concentrations in mice [213]. Together these findings identify the NO/sGC/cGMP/PKG axis as a positive regulator of erythroid maturation, though all evidence for γ-globin induction derives from human erythroid cell systems, as γ-globin is a primate-specific gene absent in wild-type mice.

The cellular source of NO regulating erythropoiesis remains unclear. Bone marrow sinusoidal endothelial cells express eNOS, suggesting that endothelial-derived NO may act in a paracrine manner on adjacent erythroblastic islands. Indeed, erythroid cells release angiogenic signals to regulate sinusoidal endothelial cells, and endothelial-derived NO may diffuse back to adjacent erythroid progenitors to activate GC-1 in a niche-dependent manner. This would represent another example of bidirectional endothelial-parenchymal crosstalk, analogous to the LSEC-hepatocyte interactions regulating iron homeostasis. However, current genetic evidence does not support this hypothesis.

A study comparing erythropoietin-induced erythropoiesis in nNOS^−/−^ and eNOS^−/−^ mice reported that only nNOS^−/−^ mice exhibit impaired erythropoiesis *in vivo* and impaired formation of colony-forming units for erythroid cells (CFU-E) ex vivo [218]. The cellular source expressing nNOS in the bone marrow is also unclear, as nNOS is most commonly associated with neuronal expression, although its expression in other bone marrow populations remains unresolved. Additionally, stromal and macrophage populations within erythroblastic islands may express iNOS under certain conditions. The relative contributions of endothelial eNOS versus other NO sources in supporting erythropoiesis thus require further investigation.

### 4.5 Integrative model: endothelial NO as a vascular-bed-specific metabolic regulator

The examples examined in this section suggest that endothelial-derived NO contributes to the regulation of organ and systemic metabolism beyond its established effects on vascular tone and perfusion. Although global eNOS- and iNOS-deficient models support a role for NO in metabolic regulation, they cannot resolve the contributions of specific endothelial populations or vascular beds. Such distinctions are likely important given the spatial heterogeneity of NO signaling. For example, in resistance arteries, endothelial α-globin scavenges eNOS-derived NO at the myoendothelial junction, limiting its diffusion to smooth muscle cells. Iron deficiency reduces this scavenging by decreasing holo-α-globin, thereby increasing local NO bioavailability. These findings suggest that endothelial subpopulations integrate local metabolic status and translate it into paracrine and systemic regulatory responses, potentially acting as metabolic sentinels within their respective vascular beds.

Endothelial-parenchymal crosstalk appears to be bidirectional. In the bone marrow, erythroid cells signal to sinusoidal endothelial cells via mediators such as VEGF and angiopoietin-1, while endothelial cells potentially signal back via NO-sGC-cGMP signaling to erythroid progenitors. This bidirectional communication enables coordinated metabolic regulation that couples vascular and parenchymal cell function with systemic homeostasis.

Loss of endothelial NO bioavailability, the defining feature of endothelial dysfunction, disrupts each of these regulatory levels simultaneously. LSEC capillarization in MASLD impairs local hepatocyte angiocrine support, promotes portal hypertension and fibrosis, and is associated with systemic endothelial dysfunction and elevated cardiovascular risk. Organ-specific differences in NO availability, as well as CD36-mediated fatty acid uptake, alter circulating lipid distribution and can contribute to ectopic lipid deposition. Whether these organ-specific manifestations of endothelial dysfunction communicate with each other through circulating factors, shared pathogenic mechanisms or direct inter-organ signaling remains an important open question. Understanding how NO flux (eNOS versus iNOS versus hypoxic NO synthesis), compartmentalization and endothelial heterogeneity intersect across vascular beds may provide the mechanistic framework for addressing vascular contributions to cardiometabolic disease.

## 5 Conclusion and outlook

This review focused on endothelial cell-derived NO as a flux- and compartment-dependent regulator of metabolism, operating through distinct mechanisms at the cellular and systemic levels.

At the *cellular level*, the source, magnitude and subcellular location of NO production determine downstream signaling mechanisms and functional outcome. Low levels of eNOS-derived NO activate the sGC/cGMP downstream pathway, elicit selective *S*-nitrosation of metabolic enzymes, modulate iron partitioning and heme insertion and support redox homeostasis. Enzymatic removal of S-nitroso groups by GSNOR and SCoR2 helps preserve the selectivity and reversibility of this metabolic regulation. Sustained, high-output iNOS-derived NO causes broader nitrosative modifications and affects mitochondrial function and insulin signaling. Protein nitration and the formation of electrophilic nitro-fatty acids provide additional cGMP-independent mechanisms linking NO-dependent redox chemistry to inflammatory and metabolic signaling. At the *organ* and *systemic level,* specialized endothelial cells emerge as metabolic sentinels within their distinct vascular beds, orchestrating endocrine and paracrine vascular and metabolic responses. However, the NO sources that govern these functions remain unresolved and require investigation in a cell-specific manner.

A central implication of this framework is that endothelial dysfunction disrupts metabolic regulation at multiple levels. It affects substrate delivery through reduced vasodilation, dysregulate cellular metabolism through altered NO-dependent post-translational modifications and redox signaling, and disrupts systemic metabolic coordination through impaired endothelial metabolic sensing. Such multilevel events may help to explain why endothelial dysfunction is central to metabolic diseases including type 2 diabetes, MASLD and cardiovascular disease, and why these conditions so frequently co-occur. More broadly, understanding how endothelial metabolic heterogeneity across vascular beds relates to NO bioavailability and function represents an important frontier, particularly as single-cell technologies reveal unexpected metabolic diversity within endothelial populations (Box 1).

From a therapeutic perspective, strategies that restore endothelial NO bioavailability, including substrate supplementation (l-arginine, l-citrulline), prevention of eNOS uncoupling (tetrahydrobiopterin) and dietary nitrate supplementation, may provide metabolic benefits beyond traditional cardiovascular endpoints. These effects, however, require more detailed investigation in human studies. Targeting specific NO-regulated pathways, such as CD36-mediated lipid uptake or LSEC-hepatocyte metabolic crosstalk, may represent a more specific alternative approach.

As mechanistic understanding deepens and new tools emerge to resolve the spatial and temporal control of NO signaling, we anticipate therapeutic strategies that address the vascular-metabolic crosstalk in cardiometabolic disease.

**Box | Open questions and future directions**

• **Human relevance of eNOS-derived NO.** The contribution of endogenous eNOS-derived NO to endothelial metabolism in humans requires further investigation, as much of the current evidence comes from mouse models.
• **NO and endothelial iron metabolism.** Evidence linking NO to endothelial iron metabolism has largely relied on high-concentration NO donors that model iNOS-associated nitrosative signaling, rather than physiological eNOS activity.
• **LSEC-derived NO in liver metabolism.** Whether and how LSEC-derived NO modulates lipid trafficking, or iron sensing and hepcidin regulation, in the liver warrants investigation — particularly given the co-occurrence of endothelial dysfunction with MASLD and with iron dysregulation in chronic diseases.
• **NO source and sGC in erythropoiesis.** The cellular source of NO and the role of sGC in regulating erythropoiesis remain unresolved, with genetic evidence implicating nNOS rather than eNOS (at least in mouse bone marrow) and only limited evidence from human cells.
• **Endothelial metabolic heterogeneity.** How endothelial metabolic heterogeneity across vascular beds relates to NO bioavailability and function represents an important frontier, particularly as single-cell technologies reveal unexpected metabolic diversity within endothelial populations.

## CRediT authorship contribution statement

**Mauro Siragusa:** Writing – review & editing, Writing – original draft, Funding acquisition, Conceptualization. **Luke S. Dunaway:** Writing – review & editing, Writing – original draft, Funding acquisition, Conceptualization. **Eckhard Lammert:** Writing – review & editing, Writing – original draft, Funding acquisition. **Ingrid Fleming:** Writing – review & editing, Writing – original draft, Funding acquisition. **Brant E. Isakson:** Writing – review & editing, Writing – original draft. **Miriam M. Cortese-Krott:** Writing – review & editing, Writing – original draft, Funding acquisition, Conceptualization.

## Declaration of competing interest

The authors declare that they have no known competing financial interests or personal relationships that could have appeared to influence the work reported in this paper.

## Data Availability

No data was used for the research described in the article.
